# Dasatinib-Induced Polymyositis-Like Syndrome: A Report of a Rare Case

**DOI:** 10.7759/cureus.86457

**Published:** 2025-06-20

**Authors:** Arathi Kulkarni, Chandana Keshavamurthy, Tamim Sultani, Divya Shastri, Trent Smith

**Affiliations:** 1 Rheumatology, Nova Southeastern University Dr. Kiran C. Patel College of Osteopathic Medicine, Davie, USA; 2 Rheumatology, University of Arizona College of Medicine - Phoenix, Phoenix, USA; 3 Radiology, University of Arizona College of Medicine - Phoenix, Phoenix, USA; 4 Internal Medicine, Creighton University School of Medicine, St. Joseph's Hospital and Medical Center, Phoenix, USA

**Keywords:** dasatinib, drug-induced myositis, inflammatory myopathy, muscle inflammation, myositis, rheumatology

## Abstract

Myositis is an inflammatory muscle disease characterized by chronic inflammation, progressive muscle weakness, and various systemic effects. Dasatinib, a tyrosine kinase inhibitor (TKI), is commonly used as a first-line treatment for chronic myelogenous leukemia (CML). Although it is generally effective, there have been rare cases where dasatinib can induce muscle damage, leading to muscle weakness. We wanted to highlight a unique case involving a 24-year-old Caucasian male who was treated with dasatinib for CML and subsequently developed proximal weakness in both his upper and lower extremities. Imaging studies, including multiple MRI scans, revealed diffuse muscular edema. Importantly, the patient did not receive steroid treatment; however, his symptoms significantly improved after discontinuing dasatinib. This case is a noteworthy contribution to the understanding of dasatinib-induced polymyositis-like syndrome, as it represents the first documented instance, to the best of our knowledge, based on our literature review. Such cases emphasize the need for close monitoring of muscle health in patients receiving dasatinib and encourage further investigation into the relationship between this medication and myositis.

## Introduction

Idiopathic inflammatory myositis (IIM) is a rare and heterogeneous systemic autoimmune disease that primarily affects the muscles and skin. Its manifestations can range from acute and potentially fatal conditions to slow-progressing chronic issues [1,2]. The recognized clinical subsets of IIM include adult polymyositis, adult dermatomyositis, juvenile myositis, cancer-associated myositis, connective tissue disease-associated myositis, inclusion body myositis (IBM), necrotizing autoimmune myopathy, clinically amyopathic dermatomyositis, and anti-synthetase syndrome [3].

Typical symptoms include symmetric weakness of the proximal muscles; however, atypical presentations have also been reported in the literature [2]. The condition may involve multiple organ systems. Pulmonary manifestations include interstitial lung disease (ILD), which may present with cough, dyspnea, palpitations, and chest pain [1]. Dermatologic findings can include various rashes that include heliotrope rash and Gottron's papules [1]. Gastrointestinal symptoms, such as dysphagia and odynophagia, may also occur [4]. Treatment typically involves corticosteroids; however, specific therapy is guided by disease severity and clinical subtype.

Drug-induced myositis can closely resemble idiopathic polymyositis, particularly in cases such as statin-induced myopathy, where patients may present with proximal muscle weakness and elevated muscle enzyme levels [5]. This may occur even after stopping the medication. Other medications, such as penicillamine and zidovudine, have also been linked to drug-induced myotoxicity that mimics polymyositis [6]. Differentiating drug-induced myositis from idiopathic forms requires a careful review of the patient's clinical symptoms, timing and duration of drug exposure, laboratory results, and muscle biopsy findings. Identifying specific autoantibodies can further assist in establishing an accurate diagnosis and management of therapy.

Tyrosine kinase inhibitors (TKIs), such as dasatinib, can cause myalgias and have been associated with myositis. Dasatinib is an oral multi-target TKI that targets the breakpoint cluster region (BCR) gene on chromosome 22, the Abelson (ABL) tyrosine kinase gene on chromosome 9, and SRC family kinases [7]. It is widely used to treat hematologic malignancies, particularly chronic myelogenous leukemia (CML), as well as certain solid tumors [7]. Reports have suggested that dasatinib has been linked to myalgias; some studies have suggested between 6% and 24% [8]. In this case, we present a patient with dasatinib-induced polymyositis-like syndrome, who showed rapid resolution in his symptoms after discontinuation of the drug.

## Case presentation

A 24-year-old Caucasian male diagnosed with CML was initially started on hydroxyurea for two weeks before initiation of 100 mg dasatinib one month after his diagnosis. The patient had a history of nicotine, alcohol, and cocaine use but reported abstinence from all substances for the past eight months. Two months after being on the TKI medication, he was admitted for a neutropenic fever. At the time, his dasatinib dosage was reduced to 50 mg. A month later, he was hospitalized with an acute onset of proximal muscle weakness in the bilateral upper and lower extremities. Additionally, there was an acute onset of a bright red rash on the anterior neck, bilateral posterior elbows, right palm, left wrist, and both the flexural and extensor aspects of the forearms and arms. There was no rash or redness on the legs, back, chest, or stomach. The rash was associated with intermittent fevers (maximum temperature of 38.4°C) and night sweats. The patient denied dysphagia and odynophagia. Dasatinib was discontinued immediately upon hospital admission.

At the time of hospital admission, the patient was found to have leukopenia, with a white blood cell count of 0.8 × 10³/µL, neutropenia at 0.02 × 10³/µL, and lymphopenia at 0.57 × 10³/µL. Additionally, the patient exhibited anemia, improving thrombocytopenia, and a blood smear revealed toxic granulations. The erythrocyte sedimentation rate (ESR) was elevated at 119 mm/hour, and the C-reactive protein (CRP) was elevated at 211 mg/dL. Fibrinogen levels were elevated to 969, and D-dimer was measured at 559. Antinuclear antibody (ANA) tests returned negative, and rheumatoid factor (RF) was 26. Liver and kidney function tests were within normal ranges. Muscle enzyme levels showed a normal creatine kinase (CK) at 39 and normal lactate dehydrogenase (LDH) at 127, but the aldolase was elevated at 12. The only source identified for the increased aldolase levels was muscle. Urinalysis revealed the presence of protein and red blood cells, though the culture results were negative.

A comprehensive infectious disease workup was performed, including blood cultures; testing for influenza, a viral respiratory panel, methicillin-resistant *Staphylococcus aureus* (MRSA), *Legionella *antigen, *Streptococcus*, *Cryptococcus*, fungal pathogens, HIV, cytomegalovirus (CMV), Epstein-Barr virus (EBV), Monospot, toxoplasmosis, adenovirus polymerase chain reaction (PCR), parvovirus, *Aspergillus *serum antigen, and *Trichinella *immunoglobulin G (IgG), all of which returned negative results. The patient did not receive antibiotics for more than two days during his admission. Extensive imaging was conducted, including chest X-rays and CT scans of the chest, abdomen, and pelvis, which were all unrevealing. Ultrasound imaging of the upper extremities did not show any blood clots. An echocardiogram revealed an ejection fraction of 56%, with trace mitral regurgitation and tricuspid regurgitation. The extended myositis panel, which tested for antibodies against 3-hydroxy-3-methylglutaryl-coenzyme A reductase (HMG-CoA reductase) and cytosolic 5'-nucleotidase 1A (CN1A), all returned negative results.

**Figure 1:**
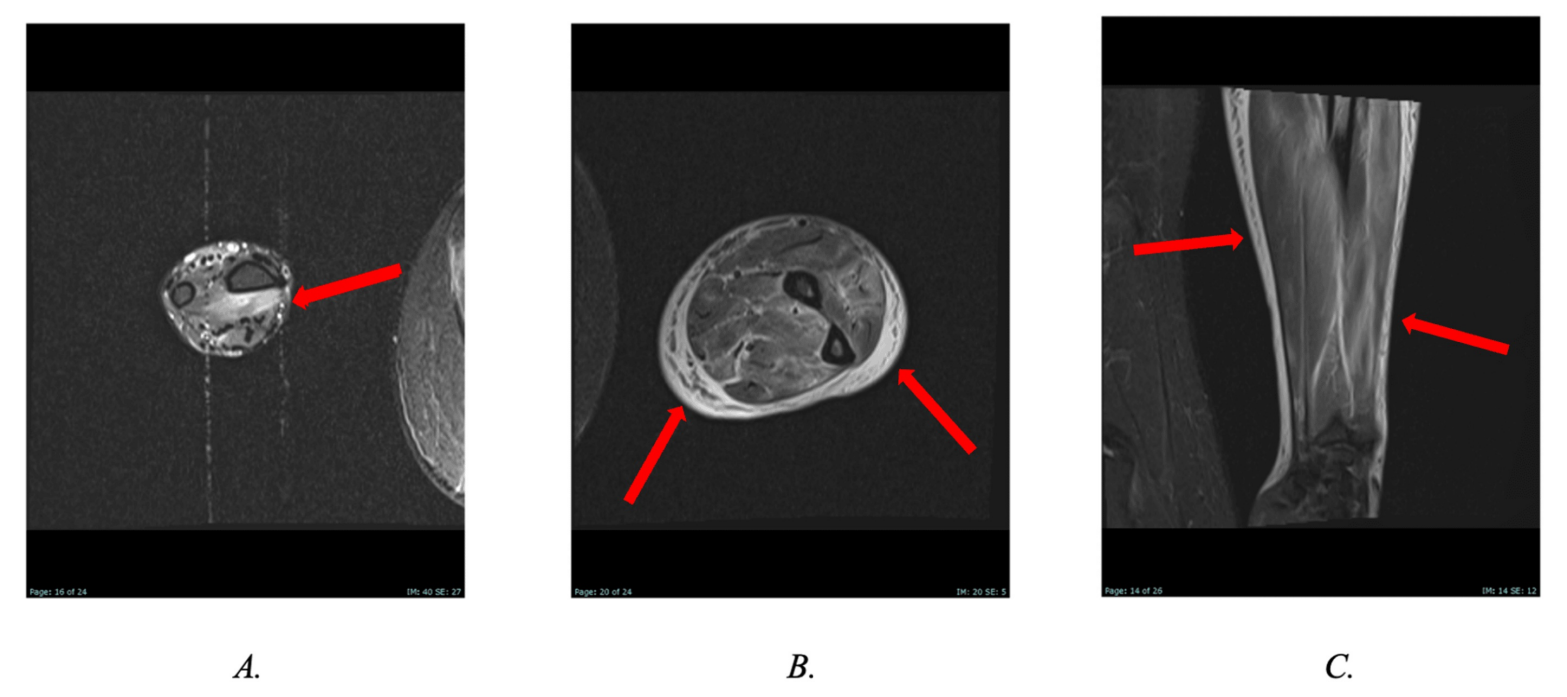
Axial STIR images (A and B) and coronal STIR image (C) of the right forearm show arrows pointing to multi-compartment muscular edema and circumferential soft tissue edema. STIR: short tau inversion recovery

**Figure 2:**
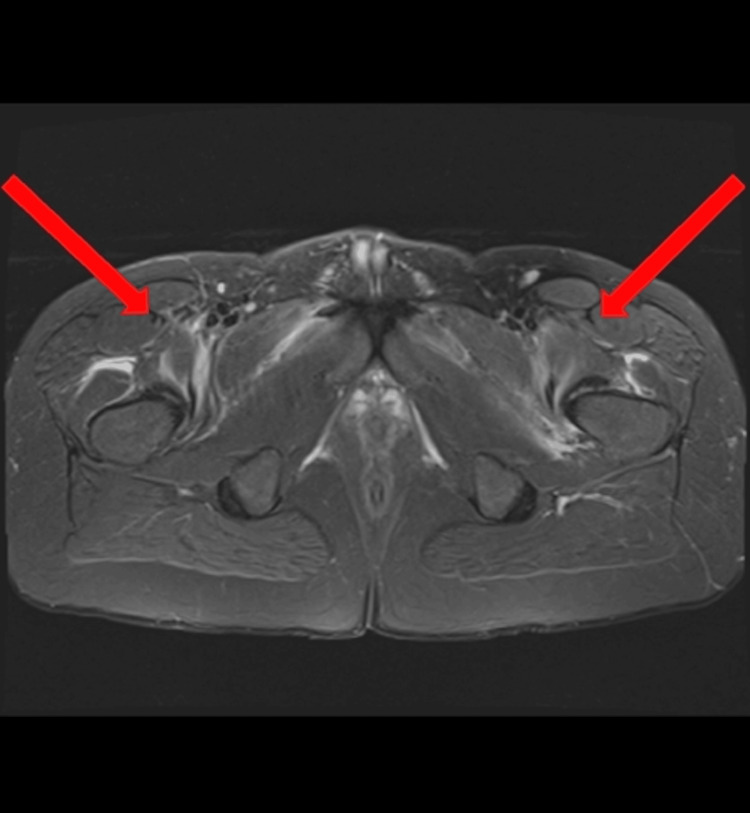
Axial STIR sequence of the pelvis showing arrows pointing to multi-compartment muscular edema, most notably in the adductor compartments. STIR: short tau inversion recovery

**Figure 3:**
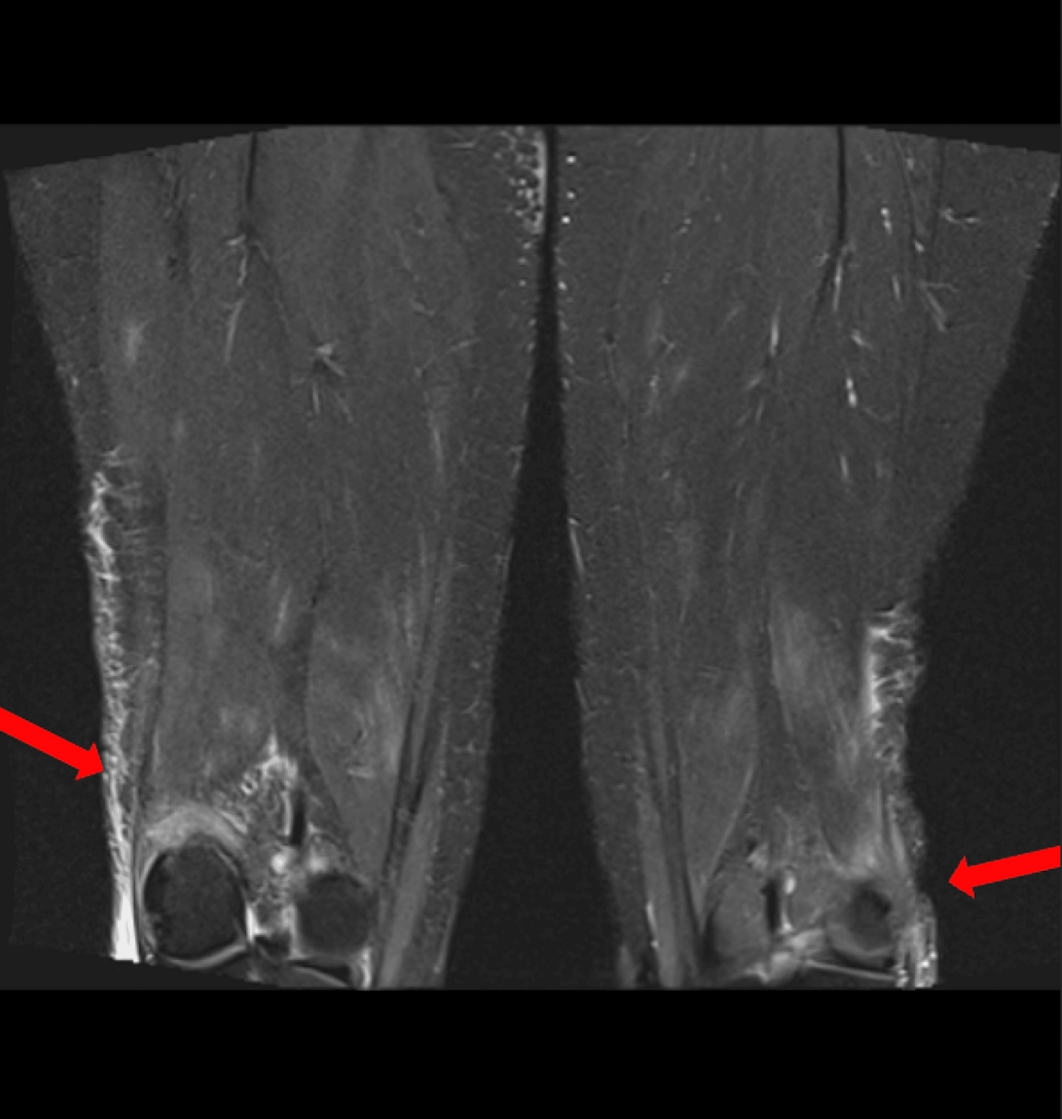
Coronal STIR images of the bilateral thighs showing arrows pointing to muscular edema and corresponding enhancement. STIR: short tau inversion recovery

Key MRI findings included mild heterogeneous marrow signal in the humerus and upper arm, consistent with bone marrow edema; muscle edema involving the biceps, brachialis, and triceps; and intermuscular edema along the fascial planes, suggestive of myositis. Additionally, the forearms demonstrated moderate heterogeneous edema of the forearm muscles, fasciitis, and subcutaneous edema (Figure 1). Bilateral adductor compartments had heterogeneous patchy muscle edema with enhancement, mild fascial edema, and no subcutaneous edema (Figure 2). Figure 3 shows bilateral lower extremities demonstrating multi-compartment edema. Five days later, follow-up MRI scans of the bilateral upper extremities, pelvis, and bilateral lower extremities were obtained, which continued to demonstrate multi-compartment muscular edema. A slight interval decrease in edema was noted in the right forearm compared to the prior MRI. Additionally, there was a reduction in muscular edema in the distal septal muscle, although scattered adjacent soft tissue edema persisted on imaging.

An electromyography (EMG) and nerve conduction study (NCS) were conducted to assess fibrillation potentials and positive sharp waves in both proximal and distal arm and leg muscles. Only the proximal muscles exhibited short-duration, small-amplitude, polyphasic motor unit action potentials and early recruitment.

After dasatinib was discontinued, the patient's aldolase levels trended down to within normal limits at 5.6. During his 11-day hospital stay, the patient's upper extremity rash and proximal weakness showed significant improvement. Based on the clinical findings and imaging, the patient was diagnosed with dasatinib-induced polymyositis-like syndrome. A muscle biopsy was considered but ultimately not performed, as the patient improved within two weeks of stopping dasatinib.

## Discussion

We present the first case of dasatinib-induced polymyositis-like syndrome, which manifested with proximal muscle weakness in the bilateral upper and lower extremities, with MRI evidence of muscle edema and enhancement with gadolinium contrast. The patient’s symptoms appeared indicative of myositis, as proximal muscles showed hyperintensity on T2/short tau inversion recovery (STIR) imaging. Notably, there was also involvement of the distal musculature - an atypical finding in most myositis subtypes. There was no evidence of trauma, infection, ischemia, compartment syndrome, denervation injury, or neoplasm to account for the observed muscle edema. The findings were most consistent with a drug-induced etiology, as symptoms improved significantly following discontinuation of dasatinib, without the need for high-dose corticosteroids. The rash's distribution was atypical for both polymyositis and dermatomyositis; however, a potential association was considered, given the concurrent onset of rash and muscle weakness. Furthermore, the rapid onset, associated pain, and prompt symptom resolution after withdrawal of the offending agent are not characteristic of idiopathic inflammatory myopathies or cancer-associated paraneoplastic myositis.

Other drugs have also been associated with toxin-induced myopathy, including cocaine. The patient had a history of cocaine use; however, the patient's reported last use was eight months ago. A urine drug screen was not completed prior to hospital admission. Of note, as much as 24% of cocaine users have been reported to have muscle injury and/or damage [9]. Patients typically present with proximal muscle weakness, worse in the lower extremities compared to the upper extremities, with laboratory findings showing elevated CK [9]. Notably, the patient did not have elevated CK on admission.

Polymyositis has been reported as a paraneoplastic syndrome in cases where CML undergoes transformation to acute myelogenous leukemia (AML) [10]. However, our patient showed no evidence of blast transformation. Myositis in patients with CML has also been associated with treatment-related effects, particularly with hydroxyurea and alpha-interferon [11,12]. Although this patient had prior exposure to hydroxyurea, the medication was discontinued well before the onset of myositis.

Another differential diagnosis considered was pyomyositis. Initially presenting with progressive muscle dull pain and fever, pyomyositis can progress to muscle abscesses and sepsis [13]. This condition can present as localized abscesses but can manifest as diffuse inflammatory or rapidly progressive myonecrotic processes [14]. In hematologic patients, pyomyositis most commonly involves the thigh muscles, with unilateral muscle involvement being more prevalent than bilateral [15]. MRI typically shows diffuse hyperintense signals in the affected muscles on fat-suppressed T2-weighted images, and both the lesions and the adjacent fascia enhance with contrast [16]. As the patient did not need antibiotics to respond and cultures were negative, a pyomyositis diagnosis was considered less likely.

Varying presentations of muscle disease have been reported with most TKIs. A case of imatinib-resistant CML who achieved molecular remission on dasatinib subsequently developed elevated serum CK levels and progressive muscle weakness. She was histologically diagnosed with IBM [17]. Another reported case involved a 71-year-old woman with CML who developed dermatomyositis sine dermatitis following imatinib therapy. She presented with proximal muscle weakness and myalgia in her lower limbs and tested positive for Mi-2 antibodies. After discontinuation of imatinib, she experienced spontaneous recovery without the need for immunosuppressive treatment [18]. There was not a consistent link between elevated aldolase levels and myositis in these clinical cases, even though aldolase elevation is commonly reported without a corresponding CK elevation in cases of myositis [19]. It remains unclear whether muscle injury results from prolonged TKI use or from the direct molecular effects of these agents [20]. TKIs are known to modulate T-cell function and cytokine signaling, potentially triggering autoimmune responses. Such immune dysregulation may lead to immune-mediated myositis, wherein the immune system erroneously targets muscle tissue [20].

## Conclusions

In this case presentation, the patient presented with bilateral proximal weakness in both the upper and lower extremities, along with a rash on the upper extremities. Initial MRI findings showed diffuse bilateral muscle inflammation, and labs revealed an elevated aldolase level. Notably, these symptoms improved once dasatinib was discontinued. Subsequent MRI sequences, taken after dasatinib was stopped, demonstrated a reduction in bilateral muscle edema and inflammation in the proximal upper extremities. Additionally, there was an improvement in the patient's proximal muscle weakness. Given the widespread use of dasatinib and its generally favorable safety profile, awareness of this rare adverse effect is essential to recognize and manage patients more effectively.
